# Quadratic Models May Provide a Useful Set of Models that Detect Combined Effects of Achievement Goals on Academic Attainment

**DOI:** 10.3389/fpsyg.2016.00029

**Published:** 2016-01-28

**Authors:** Sviatlana Kamarova, Nikos L. D. Chatzisarantis, Martin S. Hagger

**Affiliations:** Health Psychology and Behavioural Medicine Research Group, Laboratory of Self-Regulation, Faculty of Health Sciences, School of Psychology and Speech Pathology, Curtin UniversityPerth, WA, Australia

**Keywords:** achievement goals, multiple goals effect, quadratic model, combined effect, non-linear relationship

There is a consensus of opinion among proponents of theories of achievement goals that people may choose to pursue two different types of goals: mastery goals that focus on developing competence through task mastery and learning and performance goals that focus on demonstrating competence by outperforming others (Ames and Archer, 1987; Dweck and Leggett, 1988; Nicholls, 1989; Ames, 1992; Pintrich, 2000). Research in educational contexts indicates that mastery goals are reliably associated with positive outcomes such as high intrinsic motivation, high task-interest, and use of deep learning strategies (Harackiewicz et al., 2000, 2008). However, performance goals exhibit a stronger relationship with high task performance than mastery goals (Senko et al., 2011; Van Yperen et al., 2015). This is more likely to be observed when tendencies to pursue mastery or performance goals are coupled with approach reactions rather than avoidance reactions (Senko et al., 2011).

The positive effects of performance goals and mastery goals on the same or distinct educational outcomes have also lead researchers to propose that students who pursue both goal types simultaneously may experience more positive outcomes than students who adopt one type of goal only (Harackiewicz et al., 1998). The reason for this is that students pursuing both goals simultaneously may accrue the benefits associated with each goal (Senko et al., 2011). Accordingly, Barron and Harackiewicz (2001) advised researchers to examine the combined effects of achievement goals on outcomes by examining whether the following regression equation explained observations:

(1) $$\begin{array}{l} \text{A}={\text{b}}_{0}+{\text{b}}_{1}\text{M}+{\text{b}}_{2}\text{P}+{\text{b}}_{3}\text{MxP}+{\text{e}}_{10} \end{array}$$

In Equation 1, A represents individuals' achievement on a task such as the grades that students achieve in an exam. The terms M and P are individuals' responses to instruments measuring mastery goals and performance goals. The term MxP is a product term that represents the interaction between mastery goals and performance goals. The coefficient b_0_ is the intercept of the regression equation. The term e_10_ indicates residual variance unexplained by the regression equation. The coefficients b_1_ to b_3_ are unstandardized regression coefficients indicating main or interactive effects of mastery goals or performance goals on achievement.

Broadly speaking, Equation 1 supports combined effects of achievement goals on academic attainment if the main effects of mastery goals (i.e., b_1_ > 0) and performance goals (i.e., b_2_ > 0) on achievement are positive and statistically significant. Alternatively, the model represented in Equation 1 supports combined effects if the coefficient on the interaction between mastery goals and performance goals is positive and statistically significant (i.e., b_3_ > 0 ; Senko et al., 2011). However, previous research has rarely observed combined effects of achievement goals on academic attainment. A cursory review of the literature indicates that only six out of 66 studies (9%) have supported combined effects of performance goals and mastery goals on academic attainment by ways of using the model presented in Equation 1 (see Table 1). These studies confirmed an additive model. However, it can be argued that these effects could be obtained by chance, given the rather small number of significant effects. This possibility is reinforced by the fact that some of the studies exhibited borderline significant results (Finney et al., 2004; Bodmann et al., 2008; Pekrun et al., 2009).

**Table 1:** **Summary of achievement goal effects on performance outcomes**.

			**Effects**		
**Study**	**Performance outcome**	***N***	**Mastery goal**	**Performance goal**	**Mastery X Performance interaction**
Bodmann et al., 2008	Academic: final grade	121	+0.19^†^	+0.24^**^	−0.08
Church et al., 2001	Academic: final grade	297	+0.20^**^	+0.14^*^	ns
Finney et al., 2004	Academic: semester GPA	2111	+0.09^***^	+0.04^†^	ns
Pekrun et al., 2009	Academic: exam grade	218	+0.11^†^	+0.38^***^	ns
Senko and Harackiewicz, 2005	Academic: exam grade	166	+0.16^*^	+0.28^**^	ns
Senko et al., 2013	Academic: exam grade	157	+0.21^**^	+0.18^**^	ns

† *p < 0.12*.

* *p < 0.05*.

** *p < 0.01*.

*** *p < 0.001*.

*Coefficients reported only for approach types of mastery and performance goals*.

One reason that the regression model described in Equation 1 might have not been successful in detecting combined effects may be related to the fact that it does not test the hypothesis that individuals who endorse one goal at the highest possible level and the other goal at a marginally lower level exhibit optimal performance (i.e., individuals who adopt a high-mastery/moderate-performance goal profile). This is because the model implied by Equation 1 assumes that the effects of a goal (e.g., performance goal) on academic achievement are monotonically increasing within high or low levels of endorsing the other goal (e.g., mastery goal) (Edwards, 1994, 2001). As a consequence, when Equation 1 supports combined effects, it always “forces” researchers to conclude that a high-mastery/high-performance goal profile is the most adaptive goal profile. However, this statistical assumption of monotonicity may be restrictive and it may not express the ways that students who adopt both achievement goals regulate goal adoption. For example, in educational contexts, students who are inclined to pursue both achievement goals may endorse mastery goals at a slightly higher level than performance goals at the beginning of the semester when learning is important (Pintrich, 2000; Barron and Harackiewicz, 2001). Unfortunately, the model implied by Equation 1 does not test whether students who adopt a high-mastery/moderate-performance goal profile are the best performers. As a consequence, it may mislead researchers to reject a combined effect of achievement goals on academic attainment that corresponds to a high-mastery/moderate-performance profile when in fact observations support a combined effect.

A number of researchers have suggested that problems associated with Equation 1 can be overcome by applying the following quadratic equation to explain effects of goal orientations on attainment (Krantz and Tversky, 1971; Lubinski and Humphreys, 1990; Aiken and West, 1991; Cortina, 1993; Edwards and Parry, 1993; Ganzach, 1997):

(2) $$\begin{array}{l} \text{A}={\text{b}}_{0}+{\text{b}}_{1}\text{M}+{\text{b}}_{2}\text{P}+{\text{b}}_{3}{\text{M}}^{2}+{\text{b}}_{4}\text{MxP}+{\text{b}}_{5}{\text{P}}^{2}+{\text{e}}_{10} \end{array}$$

In this equation, M^2^ and P^2^ are quadratic terms that represent non-linear relationships between achievement goals and academic attainment. The coefficients b_3_ and b_5_ are unstandardized regression coefficients that represent effects associated with the quadratic terms. The model implied by Equation 2 can test a broader set of hypothesis about combined effects because the quadratic terms can examine whether achievement goals yield higher levels of academic attainment when they are endorsed at some moderate level (Griffin et al., 1999; Edwards, 2001). For example, the quadratic model may indicate that a high-mastery/moderate-performance goal profile is the most optimal profile if, in Equation 2, the main and quadratic effects of mastery and performance goals on academic achievement are linear and concave, respectively. The reason for this is that linear functions return higher levels of attainment when achievement goals are endorsed at the highest possible levels whereas concave functions indicate that achievement goals yield higher performance levels when they are endorsed at moderate levels (Edwards and Parry, 1993).

As it stands in the current literature on achievement goals, it is difficult to determine whether the quadratic model is more effective in detecting combined effects than the model implied by Equation 1. This is because the majority of studies have not included quadratic terms in their regression analyses. Most importantly, we need to explain what a curvilinear relationship in this context means. In particular, performance approach goals produce a mix of simultaneously positive (e.g., Elliot and Church, 1997; Harackiewicz et al., 1997; Senko and Harackiewicz, 2005; Hulleman et al., 2010; Senko and Hulleman, 2013) and negative or null effects on academic achievement (e.g., Ames and Archer, 1988; Greene et al., 2004; Long et al., 2007; Matos et al., 2007). On the one hand, performances goals promote academic attainment because they motivate students to actively attend to teachers' instructions, guides and hints that allow them predicting content of examinations (learning agenda hypothesis; Senko et al., 2013). These goals orient students to use studying strategies that increase probability to do well at exams. For example, allocating the studying time and effort to materials evenly ensures attainment of a high academic grade (Senko et al., 2013). On the other hand, performance goals may undermine academic achievement by increasing anxiety and perceived social pressure. For example, Eisenbarth and Petrichkoff (2012) observed a convex relationship between these variables whereby worry reached lowest levels when participants moderately endorsed performance goals. Therefore, it is possible that a moderate endorsement of performance goals yields the highest achievement, whereas any deviation (above or below a moderate level) undermines it. There is some evidence to support this relationship (Sideridis, 2007; Sideridis et al., 2013; Sideridis and Stamovlasis, 2014; Stamovlasis and Sideridis, 2014). For example, Sideridis et al. (2015) demonstrated that a mild performance class-climate increased reading performance, but more active instigation of the performance goals beyond a moderate level was maladaptive for reading performance. In addition, Sideridis et al. (2015) also observed a linear relationship between reading performance and class-climates in which teachers strongly encouraged adoption of mastery goals. Building on these findings and Senko et al.'s (2013) theoretical framework, a high-mastery/moderate-performance goal profile is expected to be the most optimal goal profile. Hence, the quadratic model may prove to be a viable data analytic model that assists researchers in detecting combined effects of achievement goals on academic attainment. We look to future research on the effects of achievement goals on academic attainment to routinely apply the quadratic model to analyse their data. This will provide definitive evidence as to whether the quadratic model is the most effective in describing interactive effects of mastery and performance goals on academic attainment.

In conclusion, the present article suggests that previous research might have not been successful in detecting combined effects of achievement goals on academic attainment because they did not include quadratic terms in their regression models. Quadratic terms, which test for non-linear relationship between achievement goals and academic attainment, enable researchers to examine a broader set of hypothesis about combined effects such as whether a high mastery-moderate performance goal profile is the most optimal. Therefore, by applying the quadratic model future research may become more consistent in detecting combined effects of achievement goals on academic attainment and provide a precise description of which goal profile is most optimal in terms of yielding highest levels of academic performance.

## Author contributions

The current paper is a part of the Ph.D. of SK. SK, NC, and MH conceived the ideas presented in the article and drafted the article. The final approval was obtained from all co-authors prior to submission.

### Conflict of interest statement

The authors declare that the research was conducted in the absence of any commercial or financial relationships that could be construed as a potential conflict of interest.
